# Three-Phase Handover Management and Access Point Transition Scheme for Dynamic Load Balancing in Hybrid LiFi/WiFi Networks

**DOI:** 10.3390/s22197583

**Published:** 2022-10-06

**Authors:** Sallar Salam Murad, Salman Yussof, Wahidah Hashim, Rozin Badeel

**Affiliations:** 1 Institute of Informatics and Computing in Energy, University Tenaga Nasional (UNITEN), Kajang 43000, Malaysia; 2 College of Computing and Informatics, University Tenaga Nasional (UNITEN), Kajang 43000, Malaysia; 3 Department of Communication Technology and Network, University Putra Malaysia (UPM), Seri Kembangan 43300, Malaysia

**Keywords:** LiFi, hybrid networks, wireless communication, energy, load balancing, handover

## Abstract

Since LiFi and WiFi do not interfere with one another, a LiFi/WiFi hybrid network may provide superior performance to existing wireless options. With a large number of users and constant changes, a network can easily become overloaded, leading to slowdowns and fluctuations in data transfer speeds. Handover (HO) increases significantly with an increase in users, which can negatively impact system performance and quality of service (QoS) due to connection loss and/or delay. Innovative three-phase handover management and AP transition (TPHM-APT) is proposed with the goals of maintaining a steady link with reduced HOs for all connected users, meeting high per-user data rates, and having low outage performance. The proposed scheme primarily focuses on reducing the total number of HOs, which improves reliability and keeps user densities low on individual LiFi APs, which conserves bandwidth and energy. Conventional methods of HO management and user assignment, such as those based on signal strength strategy (SSS), involve reallocating users to a different AP the moment they encounter a HO. Our technique consists of three stages that focus on the optical gain, the incidence angle of the receiver FOV, and user mobility speed for decision-making. Specifically, a data rate threshold (DRT), which is equivalent to the data rate gained from the optical gain, is used to determine whether users must be served by a LiFi or a WiFi AP. In addition, an incidence angle threshold (IAT) is identified to manage the handover process and user AP transition with the consideration of the user mobility threshold (UMT). The proposed method considers load balancing (LB) among all connected users as well. This approach is evaluated using Monte Carlo simulations with MATLAB. Mathematical expressions are derived to analyze the performance of the proposed method. Different aspects, for example, Outage Probability, HO Overhead, User density, System Average Throughput (SAT), and Average Data Rate Requirement (ADRR), are studied. Analysis shows performance gains in overall system performance in terms of system data rates, fairness, and HO rates. Simulation results show that against the standard HO scheme and traditional HO skipping and APA methods, the proposed scheme can effectively decrease HO rates, save LiFi resources, and increase user throughput. It also shows good correspondence to the analysis and reveals the associated trade-offs that occur when moving between the span of narrow to wide FOVs and vice versa (HO rates and APS). The proposed scheme achieves almost identical results for low-density and high-density systems as well, with different ADRR and HO overhead values.

## 1. Introduction

### 1.1. Background

In the next 15 years, worldwide data transmission is predicted to expand at a rate of 50% every year [1]. As a result, the network is under pressure to provide better service for users. With such tremendous growth, fifth-generation (5G) networks and onwards must enable high data rates, seamless connectivity, robust security, and ultra-low latency communications as soon as possible [2,3,4]. Because of its limited capacity and intensive deployment, the present wireless fidelity (WiFi) system, which is already overcrowded, will face an increased load [5,6]. To address these demands, researchers from industry and academia are experimenting with new network designs, transmission techniques, and spectrums.

To meet this rising need, the direction is toward Light-Fidelity (LiFi), a potentially critical technology for sixth-generation (6G) wireless communications [7]. LiFi is a unique bidirectional, high-speed, fully networked wireless communication technology that leverages visible light as the downlink propagation medium for illumination and transmission. It can employ infrared in the uplink to avoid interference with visible light in the downlink and to maintain the illumination constraint of a room [8]. Compared to WiFi, LiFi has several advantages, including (i) accessibility in RF-restricted places; (ii) a large and unlicensed visible optical spectrum of 300 THz; and (iii) secured communication as light does not penetrate solid walls. The data transmission from a base station (BS) to user equipment (UE) is designated as the downlink connection. A fundamental setup was shown in [9].

A modulated electrical signal is transformed into a radio frequency (RF) electromagnetic wave output in RF systems through an RF chain. In LiFi systems, on the other hand, transmitter front-end components transform a modulated electrical signal into a light signal, and reception front-end units transform the transmitted light signal back to the modulated electrical signal. The majority of low-cost incoherent LiFi front-ends find that intensity modulation (IM) with direct detection (DD) is adequate for downlink broadcasting. The adoption of IM/DD methods influences the parameters of the downward transmission route in part. On-off keying (OOK), pulse position modulation (PPM), pulse-amplitude-modulated discrete multi-tone modulation (PAM-DMT), and optical orthogonal frequency-division multiplexing are some of the possible modulation methods (O-OFDM) that could be used in LiFi systems [10].

A LiFi access point (AP) primarily serves a limited region of around 2–3 m in radius. As a result, mobility management issues that result in horizontal handover (HHO), which occurs within the domain of a single wireless access technology [11], where the connected user is relocated to another access point, are unavoidable. These regular handovers (HO) boost the likelihood of connectivity interruption and ultimately result in packet losses and delays, resulting in a bad user experience. Another key element that causes handovers in LiFi networks is light-path obstruction [12].

Hybrid LiFi and WiFi networks were established to merge the high data rate of LiFi and the prevalent coverage of WiFi in an attempt to alleviate the aforementioned problems [13] since LiFi is complementing the WiFi network, establishing a hybrid networking system. Vertical handover (VHO), on the other hand, takes place between several wireless access technologies, such as WiFi and LiFi, or vice versa, in this hybrid system. A VHO typically adds a significant delay compared to an HHO as a result of different medium access control pathways [14].

**Figure 1:**
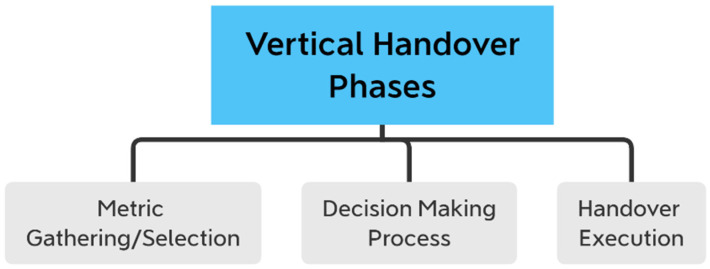
The structure of VHO.

As a result, repeated VHOs would drastically lower system capacity. Similar to other hybrid networks, maintaining signal continuity requires corresponding HO techniques and algorithms. The VHO algorithm can be designed using any mathematical techniques that can be used to fit a multiple-input, single-output function, which is how the method is defined mathematically. Figure 1 [14] illustrates how a VHO process can be broken down into three parts, including metric gathering, decision-making, and handover execution modules. Decision-making, also known as the HO algorithm, is the most significant step. Previous VHO algorithms can be divided into several groups depending on the mathematical techniques employed, including fuzzy logic (FL), Markov decision process (MDP), game theory (GT), and machine learning (ML)-based algorithms [14]. It is necessary to simulate the algorithm to assess its performance after choosing the appropriate mathematical tool to model the VHO problem.

Hybrid networks have the potential to raise network performance, though when user mobility is factored into the equation, load balancing (LB) concerns and the consideration of correct HO decisions become more difficult and complex. This is because the WiFi AP typically has a lower capacity than the LiFi AP [15] but a wider coverage area. To prevent inter-cell interference in a homogeneous network, the overlapped coverage among LiFi APs must be abridged, then the LB process would not be necessary. As a result, the signal strength strategy (SSS), which places every user with the AP that has the strongest received signal strength, is frequently utilized in homogeneous networks. As a result, if the SSS approach is employed, a WiFi AP would be able to serve more users than a LiFi AP. The WiFi system becomes vulnerable to traffic congestion as a result. Therefore, employing the SSS would easily cause the WiFi AP to become overloaded. LB with VHO and HHO is necessary for the reasons listed above.

The term “Attocell” refers to the LiFi AP’s coverage region. Co-channel interference (CCI) or optical CCI is produced when LiFi attocells cross paths with one another. This is considered extra noise in [16]. The feasible spectral efficiency in the LiFi systems would be severely impacted by the CCI when LiFi APs are placed extremely close together. Depending on the system criteria to be satisfied, an efficient solution should be employed to solve this problem if the distance between LiFi APs is not specified to be more than the size of an attocell. Users would connect when they are inside the service area, which is represented by a LiFi attocell. Attocell size and illumination, however, are influenced by a variety of factors, including:

- The distance between the transmitter in the AP and the receiver;
- The height of the room; and
- Optical energy transmission.

LiFi APs provide service in connected areas that are often smaller than LiFi attocells as a whole. These areas are specifically non-circular in the optical CCI scenario but circular in the non-CCI situation, and that could make the access point selection (APS) process complicated. A hybrid LiFi and WiFi network generally creates APS problems, and users occasionally may experience HO even when they are inside the cell for the following reasons:

- The fluctuations of the data rate of the LiFi AP;
- The received optical gain;
- The variation of the HO circle caused by the variations of RF data rate;
- A WiFi AP dominantly attracts the users close to it, leading to inefficient use of nearby LiFi APs [17];
- A WiFi AP has a larger coverage area but less capacity than a LiFi AP, and thus is more susceptible to overload; and
- User navigation including direction and speed.

**Figure 2:**
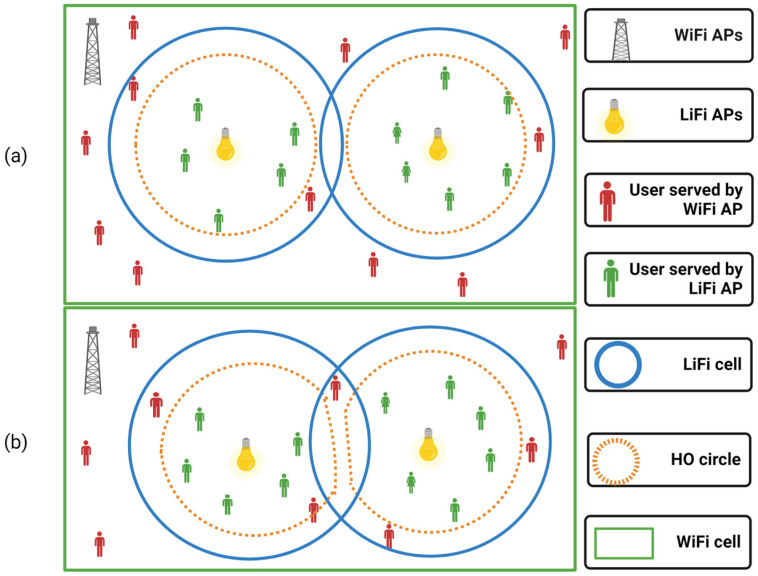
HO Circle Illustration. (**a**) HO circle does not overlap, (**b**) HO circle overlapped with another.

Users served by LiFi APs must dwell within the HO circles, as indicated in Figure 2a. Users linked to the related LiFi AP do not encounter optical CCI when a HO circle does not intersect with other attocells. Moreover, as illustrated in Figure 2b, which also depicts the boundaries of the service area of LiFi APs, part of the users handled by this LiFi AP will face optical CCI when a HO circle overlaps with other LiFi attocells. The arrangement of the LiFi APs, however, has a major impact on the precise geometry of the LiFi service area in the optical CCI situation. Users serviced by LiFi APs do not receive any optical signals from other LiFi APs in the non-CCI condition. Each LiFi AP is supposed to cover an attocell of the same size. Any two LiFi APs should be farther apart than the diameter of LiFi attocells to eliminate optical interference.

### 1.2. Related Works

In LiFi networks, a sizable amount of research has been done on LB, APS, HHO, and VHO. A thorough comparison of instant VHO (IVHO) and dwell VHO was reported in the study [14], termed (DVHO). When the received signal strength (RSS) or the SINR received from the optical channel is less than a predetermined threshold, the IVHO is the approach used to perform the VHOs to RF networks instantly. In other words, with low arrival data rates, it will complete the HO with reduced wait time. However, when the arrival data rate rises, this benefit gradually disappears and may even start to work against it. As a result, this strategy will result in too many ping-pong switches between the two networks, which will increase latency and signaling costs. Mobile terminals (MTs) are made to wait for a dwell period before performing the HO to prevent this. The VHO procedure will start once the VLC channel is discovered to be interrupted, and a predetermined dwell value will be kept in the dwell timer. The MT will remain in the VLC networks if the optical connection returns before the dwell duration expires; otherwise, the HO to the RF network will be carried out. In contrast to the DVHO, IVHO-MTs will instantly switch to the RF network if the optical network is discovered to be inaccessible; in other words, the dwelling value is set to zero. It is significantly simpler to switch from the RF to the VLC network. As soon as an optical channel becomes available, MTs on RF networks will migrate back to VLC ones since the VLC network can always offer a higher level of service (QoE).

The dynamically LB system in [16] attempts to address the HO problem by accounting for user mobility and handover overhead (HHO). The study, however, did not take into account lowering HO rates; instead, it used a straightforward user mobility strategy in which fixed users are served by LiFi attocells and moving users are serviced by a WiFi AP. In addition to user mobility, HO management and HO skipping are significant factors that influence the LB process. The stability difficulties in an indoor hybrid network must be taken into consideration because an HO may result in an additional HO [16].

User activities have a significant impact on LB, HO, and APA, especially in extremely congested networks [18]. Numerous research studies have been conducted to address the LB problem. In a previous study [19], the LB problem was stated as an optimization problem using proportional fairness allocation of resources. To simultaneously solve load balancing and power allocation, the authors of [20] presented an iterative approach.

With single transmission (SM) and multiple transmission (MT), the concept of mobility-aware LB was first introduced in [21] to deal with HOs brought on by user mobility. The VHO was avoided in the MT mode by serving each user simultaneously through LiFi and WiFi, so a joint optimization problem was formulated to balance traffic loads between LiFi and WiFi in this mode. Nevertheless, the user selection process (USP) for the AP was based solely on the signal strength strategy SSS in both the ST and MT modes, despite taking obstructions into account. Additionally, the USP procedure was not taken into account in the MT mode because the user is connected to both networks. The study recommended serving the user through the LiFi network to conserve WiFi resources, which means the traffic load on LiFi will increase as user data rate requirements rise. This is another restriction of the MT mode.

Optimization-based techniques frequently demand a significant amount of computing energy. This inspires the creation of rule-based methodologies [17,22]. Specifically, a fuzzy logic-based APS method that can reach near-optimal performance with a large reduction in computational cost was proposed in [17], and game theory was used in [22] to modify each user’s APS approach. Nevertheless, rule-based techniques would be less adaptable to alterations in network configuration. Furthermore, in the second stage of the fuzzy logic process, where the study eventually generated FL-SSS and FL-LB, the APS approach employing fuzzy logic in [17] relies on the traditional SSS and LB methods. Even though this method lowered processing energy, the FL-LB attained greater complexity than the FL-SSS. Optimization-based LB methods with extremely significant computing costs were developed [19] to link AP selection with the allocation of resources. Researchers have tried to use fuzzy logic [17] and game theory [22] to decrease processing energy. However, the work in [17] can simply offer inadequate answers, whereas the work in [22] needs a lot of iterations to reach a steady state. Additionally, the authors of [17,19,22] did not take user movement into account.

Researchers have been attempting to use machine learning to improve LB. Learning strategies for updating the probability distribution of each AP for making APS judgments were proposed in [23]. The reward function of the reinforcement learning technique used by the authors in [24] to balance traffic loads is intended to maximize long-term average network throughput as well as user satisfaction and fairness. In addition, an HO system was created [25] based on artificial neural networks to modify the selection preference between LiFi and WiFi.

In two studies [26,27], the authors presented soft HO techniques to maintain high data rates while moving. VHOs, however, were not taken into account. Due to the required wireless interface switch, VHOs have a higher OH. Other LiFi network investigations that have been conducted were centered on VHO techniques. In another study [28], the likelihood of VHO for a user rotating at random in hybrid RF-LiFi networks is examined. Also suggested for enhancing VHOs is the Markov decision procedure [29]. In previous work [30], a different VHO strategy that forecasts the parameters for access delays, data volume, and interruption length was put out. The system uses these criteria to decide when to transfer sides. A study [31] also looked into the effectiveness of mobile hybrid RF-LiFi hotspot networks. To address line-of-sight (LOS) blocking, fuzzy logic-based VHO techniques were presented [32].

The idea of HO skipping was established to prevent frequent HOs in the extremely crowded network [33,34,35]. In the work presented in [33], a topology-aware skipping technique was put out, which involved tying the chain length of the cell to a predetermined threshold. A similar approach, which expands the work to the multi-AP association, was reported in [34]. According to the user’s velocity, the authors of [35] created a velocity-aware handover technique that employs the best connected, Femto bypassing, Femto neglect, and macro skipping approaches. The techniques in [33,34,35] depend on the user’s itinerary being known. As a consequence, they have the following drawbacks: (i) in an indoor environment, the measurement of the user’s trajectory is less accurate due to uncontrollable inaccuracies brought on by numerous surface reflections, and (ii) additional input is required to transmit this information to the AP. Another study [36] suggested a unique HO skipping strategy based on reference signal received power (RSRP) that negates the need for extra input to address this problem. The method determines the HO target by combining the RSRP’s value and rate of change. The new technique makes use of the rate of change in RSRP to determine if a user is moving toward the AP’s center. The suggested technique determines whether or not to skip a specific AP by calculating a weighted average of the value of RSRP and its rate of change. The problem of varying and fluctuating loads on APs, as well as interference, could, however, have a significant negative impact on relying on the RSRP [37]. The study also only took into account LiFi networks.

The authors of [38] also worked on movement control in multi-tier LiFi networks utilizing randomized geometry in conjunction with the dwell time technique. Closed-form formulas for the cross-tier HO rate, ping-pong rate, and sojourn duration were generated in terms of three factors: (i) the received optical signal intensity (ROI), (ii) time-to-trigger (TTT), and (iii) user mobility. They employed distinct circumstances of semiangle (30 and 60) at half illuminance of the primary and secondary cells and provided three alternative scenarios for semiangle at half illuminance. The objectives of their study are summarized as follows:

- The study’s first goal was to lower the rate at which mobile users switch from the primary AP to the secondary AP, which is determined by how often a primary-tier UE crosses the secondary tier’s coverage boundary. In this paradigm, the value of the ROI is used to determine when to HO control.
- The second goal of the model was to lower the ping-pong rate. The key aim of this model was the HO procedure from the secondary AP to the primary AP, which stands based on a unit time metric of the user within the small cell’s coverage area for a shorter period than the time-of-stay threshold.

Very few restrictions can be inferred from their work: (i) the study suggested the secondary AP is 1.2 m height with indoor area for mobile users with 1.4 m/s velocity, which is not realistic because, in this scenario, the ping-pong effect will occur as long as the service area is too tight; (ii) the work consisted of primary LiFi AP and secondary AP and did not consider the users’ QoS; (iii) the study did not take into account any kind of hybrid LiFi system; as a result, only HHO was taken into account and without HO overhead, making it appropriate for a particular situation and environment stand-alone LiFi network; and (iv) most importantly, LB among multiple users was not taken into consideration. However, as they noted in their conclusion, “The findings will be valuable in practical LiFi deployment and planning as well as handover optimization in ultra-dense networks”. The principle they present is very helpful for developing a future HO scheme in a full LB framework/scheme.

As explained earlier, only a few studies proposed HO skipping and management, but most of them consisted of different factors. The following Table 1 summarizes the closest works including important factors that aim to compare them with our work.

The most main details are introduced, including a summary of the study’s goals and suggested methods/techniques, as seen in Table 1. Many research studies took mobility into account. User density was also taken into account while evaluating performance, but no study used it to determine whether to use APA, load balancing, or HO management. Only a few research took into account HO overhead, and LB was one of the key areas of study. Both types of HOs have been investigated in the literature and should be mentioned. Nearly all research used simulation-based implementations to assess the effectiveness of their methodology. The findings and results of each investigation are displayed in the table. In addition, additional aspects are offered, including challenges and problems. Some of these additional factors are discussed as follows in the preceding table:

- Mobility: user movements and/or speed across the coverage area of different APs.
- Blockage: some studies considered blockage as a primary aspect of the process of APS and/or handover, while some others considered it as a secondary aspect for a specific situation,
- Interference: different types of interference were considered such as inter-cell interference ICI. Most studies considered the signal-to-interference-plus-noise ratio SINR, while other studies considered a signal-to-noise ratio SNR where interference was treated as additional noise for both.
- User density: the number of users was considered to show the performance of the system against the used metrics such as system throughput, data rates, CDT, handover rates, etc.
- Load balancing: it can be seen that most load balancing studies considered also handover and APS with blockages and/or user mobility.
- Implementation: the most popular implementation method was using simulation including Monte Carlo simulation using MATLAB software.
- Handover type: most studies considered VHO since it exists in most hybrid LiFi networks.
- Topology: it can be seen that some networks in this section are VLC but not LiFi. The reason for choosing these studies is that the methods and/or scenarios in these studies including the concepts were very similar to those in LiFi in terms of APS and HO.
- Handover overhead: only a few studies considered the handover overhead in hybrid LiFi networks.

By summarizing previous works, we found that no study has studied the LB problem in hybrid LiFi/WiFi networks with reduced HO rates that considers the user density issue and resource-saving with HO overhead consideration and mobility. To the best of the authors’ knowledge, this issue has not been addressed so far.

### 1.3. Motivation

By adapting current lighting networks, a LiFi network attocell can be developed; it is anticipated to have all of the features of a cellular system. As a result, there are still many significant research questions related to the LiFi attocell network that need to be answered. These include questions about downlink and uplink transmission, interference mitigation strategies, multiple access strategies, mobility assistance, HO schemes, and backhaul connections. In this section, the importance of HO management, the impact of user density on the system, the fluctuations of resources, user mobility, the relation between user mobility and HO, the relation between the QoS and receiver FOV, and the impact of narrative FOV are discussed.

When a UE enters the coverage area of a nearby AP after leaving the coverage area of an AP, HO is typically necessary. Frequently, HO is also necessary because the transmission channel has been badly interfered with, faded, or is overloaded in the current cell. A UE may experience frequent HO, which results in throughput loss and poor user service quality if it is covered by a low-tier AP while traveling at the edge of the low-tier AP coverage region. To minimize needless HO, the UE should be moved to a higher tier AP with wider coverage. It is anticipated that as cell size is reduced, HO frequency for moving UEs would rise. An increase in HO sessions reduces system throughput and lowers service quality.

In comparison to other kinds of small cells, this HO problem is more urgent and might be thought of as the tiniest cell. However, because the optical base stations are physically close to one another, backhaul restrictions for centralized control are implied. Since LiFi networks have a high HO frequency, soft HO was taken into account by the majority of HO studies for improved quality of service, or QoS, which refers to the general communication efficiency that LiFi users experienced. The load will decline as users are disconnected from a particular AP.

According to the study in [16], resources are freed up to increase data rates for current customers as a result of the LiFi attocell load decreasing. Therefore, the goal is to create LB schemes for a hybrid LiFi/WiFi system that guarantees high user throughput, low handover overhead, fairness, and stability. When considering device movement, the study also noted that users will experience multiple HOs in standard systems as they move from one cell to another. During HOs, signaling information is exchanged between users and the central unit (CU), and depending on the algorithm used, this process can take anywhere between 30 and 3000 milliseconds on average. This implies that data loss over this period is possible, particularly when users encounter VHO, in which users are switched between networks. According to the study [16], user movement may result in local overload conditions that prevent HO and impair performance. As a result of the transmission loss, the HO overhead may result in an offset in the HO location.

The results of the study in [13] demonstrated a saturation trend of system aggregate throughput as user density rises. Due to the full utilization of the LiFi resource, while users are moving, their suggested dynamic HO system offers significant throughput increases over the static system in the event of low user density. However, when the user population grows, the gains diminish and sometimes even turn negative. This is primarily due to the impact of HO overhead. The study suggested that the optimal resource distribution in the system should take into account the proportion of mobile to fixed users. Moreover, a study [23] also found that the state of the system fluctuates depending on how many users are served by each lamp and that it is challenging to obtain the precise state information at each decision-making time instant in a distribution of LEDs that is so dense in the face of a dynamic environment.

The findings of another study [14] state that the dwelling value of the DVHO algorithm affects how effectively it performs. On the one hand, a dwell time that is too short will not completely prevent the ping-pong effect, while a dwell time that is too lengthy will add delay. The results of this study motivate others to create a novel VHO scheme with a dynamic dwell value that can adapt to various working scenarios and channel characteristics. This VHO method is anticipated to continue keeping track of several variables, such as channel conditions, user mobility, and arrival data rate. The HO choice and the ideal dwell value for the following interruption event will be made using real-time data. Future research on novel HO algorithms can benefit from these discoveries. In terms of reducing the amount of VHO, the mobility-aware load balancing in [21] has produced excellent results, despite the expense of an elevated HHO rate.

As discovered in a study [22], the QoS of users in a standard hybrid LiFi/RF system declines as the data rate demand rises. This indicates that as more users are assigned to LiFi APs, this decline in QoS is a result of the higher data rate requirements. In addition, the study [22] concluded the following: (i) the blockage aids in increasing the system’s total data rate [20], (ii) average user QoS drops with an increase in the maximum vertical receiver orientating angle when the receiver field of view is 90 degrees, (iii) the QoS performance increases as the FOV of the LiFi receiver reduces, (iv) LiFi data speeds will decrease due to a severe route loss caused by a big maximal vertical ROA, (v) when the field of view is 45 degrees, the QoS first rises before falling as the receiver orientation angle rises, (vi) LiFi receivers are perpendicular to the ground when the vertical ROA is equal to 0, the angle of irradiance is equal to the angle of incidence in the LoS optical channel, and (vii) the user QoS can be enhanced by using a modest FOV and a correct vertical ROA. Take note that in the majority of current hybrid LiFi research, the LiFi receiver FOV is 90. Additionally, the application of the RSSI for HO in a study [28] found that doing so does not ensure that the randomly-oriented UE is always connected to the closest AP.

Therefore, the above statements, findings, and facts support the motivation for this study, and that to improve QoS, it is critical to investigate and address the APS issue that is brought on by HOs while taking into account the strain that user density places on each AP. Major restrictions exist in the LB techniques currently used. These techniques emphasize increasing network throughput in bits, however, the majority of them do not take other limits like QoS measurements into account. In reality, network services, which may have varied demands for data rate, require a specific QoS level to be supported. Maximizing network throughput does not necessarily translate into lower HO rates. Therefore, the proposed technique in this study focused on the importance of resources, user density, system throughput, and HO rates. Moreover, the above discussion supports the use of user mobility and receiver FOV that were used in the proposed technique.

### 1.4. Contribution and Outline

To ensure a stable connection for all users with minimized HO rates to satisfy a high per-user data rate while achieving minimum outage performance, a novel three-phase handover management and AP transmission TPHM-APT is proposed to minimize the number of handovers and reduce user density on each LiFi AP, and perform Access Point Transition APT to ensure stable and high-quality connection for selected users for saving LiFi network resources and reduce HO rates. The proposed method considers load balancing as well. Mathematical expressions are derived to analyze the performance of the proposed method.

Furthermore, a data rate threshold (DRT) is used, which is represented as the data rate achieved from the optical gain to identify whether users should be served by a LiFi AP or a WiFi AP. In addition, an incidence angle threshold (IAT) is identified to determine the management of the HO process and user AP assignment with the help of the user mobility threshold (UMT). Different aspects, for example, Outage Probability, HO rates, HO area locations, user density, user mobility speed, Average Throughput of the system (SAT), HO overhead, and Average Data Rate Requirement (ADRR), are taken into account. Moreover, this study focuses on the relationship between user density and HO rates.

The average throughput outage probability and HO probability are investigated, as well as the link between the three thresholds and the HO probability and SAT. Aside from the limited-service area of LiFi, the challenge of mobility management is made more appealing by the disparate distribution of light intensity by various light sources. Monte Carlo simulations are used to evaluate this technique. The analysis reveals overall system performance enhancements in terms of system data rates and HO rates. The simulation findings show that, when compared to the normal HO scheme and traditional HO skipping, the suggested approach efficiently reduces the HO rate, saves LiFi resources, improves system reliability, and promotes user throughput.

The paper is organized as follows. Section 1 shows the introduction, including background, related works, motivation, contribution, and outline. Section 2 explains the methodology, including system setup, channel models, and the proposed scheme. Results and discussion are given in Section 3. Finally, the conclusion of this work follows in Section 4.

## 2. Methodology

### 2.1. Introduction

The installed LED lamp’s output shall meet the interior illumination standard as well as the photobiological safety standard [39,40]. A typical LED chip has an optical energy of a few mW, which is far lower than the value required for normal lighting functions. Multiple small LED chips should be integrated to produce an LED array, which is comparable to a single high-energy light source, to build a lamp with thousands of lumens. This approach is used in the majority of high-power LED devices [9]. Many detailed criteria, such as color appearance, unified glare rating, illuminance level, uniformity, retinal blue light hazard exposure limit, retinal temperature exposure limit, and so on, should be considered in operation. These measures’ values must be kept within the allowed limit. It is unnecessary to analyze all of these metrics in this study because they are not of primary importance in a communication system. A few of the most important key metrics in communication are:

- The lamp’s optical output, which defines the maximum energy of the forwarded signal, and
- The radiation characteristics determined by the transmitters dictate the distribution area, the area of the attocell, and the angle of irradiance φ.

**Figure 3:**
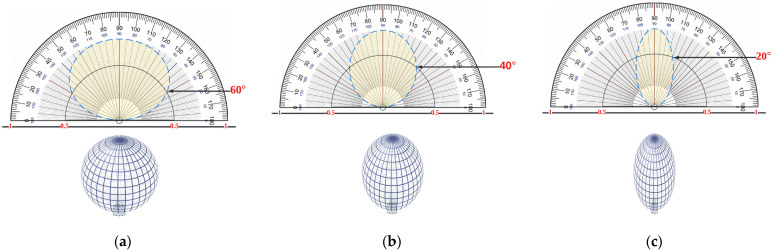
2-D and 3-D views of LED source radiation patterns, (**a**) φ1/2 = 60°, (**b**) φ1/2 = 40°, and (**c**) φ1/2 = 20°.

As a result, as illustrated in Figure 3, a simple method is employed to estimate the light output that the lamp can offer.

This section discusses the development technique used in this study. A dynamic load balancing technique is proposed in this research approach to discover the optimum AP assignment to satisfy specific average per-user data rate limitations while attaining minimum outage performance. For the downlink, an indoor LiFi/WiFi hybrid network with numerous LiFi and WiFi APs is adopted. The system and channel models, as well as the protocol and equations, have been thoroughly defined. In this unique method, three variables are used in the decision-making process, which are outlined below:

- A data rate threshold (DRT) is used to determine if a certain user gets serviced by a LiFi AP or a WiFi AP, and it is expressed as the data rate obtained from the optical gain and is denoted as γ.
- An incidence angle threshold (IAT) is identified to determine the management of the HO process and user AP assignment with the help of the user mobility threshold (UMT), both thresholds are identified as Ϯ and ⱱ, respectively.

**Figure 4:**
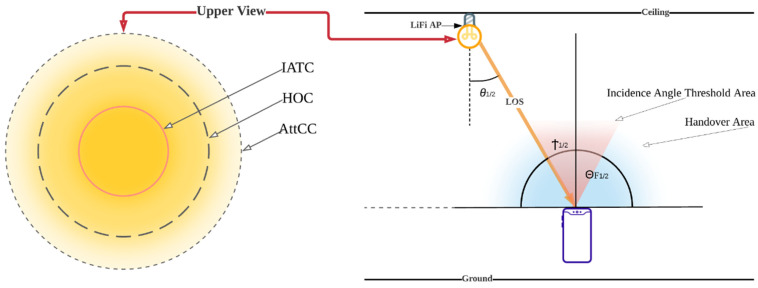
Illustration of the HOC inside the attocell with incidence angle threshold area IATC and AttCC.

Figure 4 shows the HO circle (HOC), which refers to the service area with high optical gain; the attocell circle (AttCC), which is a critical area with low optical gain; and the incidence angle threshold circle (IATC) where the highest optical gain resides. All these elements are considered in this study for the decision process of the proposed method including DRT, IAT, and UMT, as will be discussed later in this section.

The average throughput outage probability is investigated, as well as the link between the HO overhead and the three criteria. Furthermore, given the outage limits, the ideal threshold for achieving the highest average throughput for each user is determined. As a HO may prompt other HOs, the stability issue is considered. Furthermore, as the strain on the LiFi attocell decreases, resources are anticipated to be freed up to improve data rates for present users. As a result, the relationship between user density and HO rates is being explored in an attempt to comprehend the influence of different user densities on HO rates and system data rates. The computer that runs the MATLAB software to implement this project is a Lenovo Laptop with the specifications mentioned in Table 2.

### 2.2. System and Channel Models

For the downlink, an indoor LiFi/WiFi hybrid network with multiple LiFi and WiFi APs is considered. *N_v_* and *N_r_*, represent the number of LiFi and WiFi access points. Each LiFi AP is made up of an LED light made up of multiple LEDs. All photon detectors (PD) in the mobile are considered to be straight to the ground (this means the angle of irradiation is equal to that of incidence).

Furthermore, because the walls of a room entirely exclude light, co-channel interference (CCI) between rooms is prevented. As a result, under this approach, each LiFi AP covers a limited cell. Users who live in the overlapping area of LiFi attocells and are serviced by the LiFi APs will experience CCI because all of the LiFi APs consume the same bandwidth. CCI is taken into account in this study and is handled as additional noise. Users in this network are spread at random across the service area. Because of the bandwidth reuse of LiFi APs, the LiFi system may provide users with excellent spatial-spectral efficiency. However, mobile users’ optical channel state information (CSI) varies within the service region, and optical channel gain might be very poor when light rays are obstructed. A WiFi network is added to the system to boost user data rate capability.

First, it is assumed that users with strong optical channel gains are assigned to LiFi APs in this approach to better use LiFi’s excellent spatial spectrum efficiency. WiFi access points serve users with low optical CSI. WiFi access points serve users with low optical CSI.

Because of the portability of the receivers, the CSI of combined LiFi and WiFi communication lines fluctuates, requiring resource allocation to be done frequently within specific intervals of time. In this study, it is believed that user CSI fluctuates slowly, implying that CSI persists for a short length of time. As a result, the system’s operation can be separated into multiple states in relatively small periods. It is believed that the WiFi APs will cover the full interior setting. In the concept, a central unit (CU) is supposed to continually observe the system at intervals of time equal to the actual layer’s frame period.

The period of time *Tp* is described here as a situation in which all users receive allocation results from the CU and signals from APs at consistent data rates. The state’s pattern number is given by a natural number *n*. A HO occurs during user mobility when a user is served by two separate APs in two neighboring states, and the overhead is taken into account. A novel LB algorithm for operation within every state is presented in this work.

The proposed algorithm contains APT, HO decision, and transmission resource allocation. Users having a LiFi data rate of more than γ are assigned to LiFi APs, while others are assigned to WiFi APs. Furthermore, the hybrid system requires an averaged data rate for each user, represented as Г. During the working time, each user’s obtained averaged data rate ought to be greater than these criteria. The goal of this study is to determine how the threshold influences the ADRR outage. The incidence angle threshold Ϯ and the user mobility threshold ⱱ are then used to govern the APT and HO processes.

Here *N_u_* is the number of the users; *N_s_* is the number of the working states; *C* ℒ = {*v*|*v* ∈ [1, *N_v_*], *v* ∈ Z} is the set of optical attocells; and *C Ʀ* = {*r*|*r* ∈ [1, *Nr*], *r* ∈ Z} is the set of WiFi cells. The following Table 3 shows all notations and symbols used in this study.

The optical channel gain of a LiFi line of sight (LOS) channel is defined as follows:

(1) $$H=\{\begin{array}{cc} \frac{\left(\left(m+1\right)A_{p}\right)}{2\pi 〖{(z〗}^{2}+h^{2})}g\left(\theta \right)Ts\left(\theta \right)\cos^{m}\left(\phi \right)\cos\left(\theta \right), & \theta <\Theta_{F},\\ 0, & \theta >\Theta_{F}, \end{array}$$

where *m* is the Lambertian index, that is a parameter of the half-intensity emission angle *θ*_1/2_, given as *m* = −1/log_2_(cos(*θ*_1/2_)); *A_p_* is the receiver’s physical area of the photo-diode, z is the horizontal distance from a LiFi AP to the optical receiver, *h* is the height of the room, Ts(*θ*) is the gain of the optical filter, *θ* is the angle of incidence, ϕ is the angle of irradiance, $\Theta_{F}$ is the half angle of the receiver’s FOV, and g(*θ*) is the concentrator gain:

(2) $$g\left(\theta \right)=\{\begin{array}{cc} \frac{x^{2}}{sin^{2}\Theta_{F}}, & 0\leq \;\theta <\Theta_{F}\\ 0, & \theta >\Theta_{F} \end{array},$$

where *x* is the refractive index. The LED bulbs in a LiFi system operate in the linear area, where the output optical energy is proportionate to the input voltage. In addition, intensity modulation and direct detection (IM/DD) are utilized to ensure that only correct real-valued signals are sent to receivers. Before LiFi transmission, a DC bias voltage source DC is applied to the modulated electric signals. The next formula governs the conversion of the median electric energy of signals to average optical energy:

(3) ı=PoptPt

where in *P_opt_* is the median broadcast optical power of the LiFi AP α, which is proportional to *κ*_DC_, and *P_t_* is the signal’s electric power. The signal-to-interference-plus-noise ratio (SINR) for a given user *μ* linked to a LiFi AP is expressed as:

(4) $$SINR_{\mu ,\alpha }=\frac{{\left(\kappa P_{opt}H_{\mu ,\alpha }\right)}^{2}}{\iota^{2}N_{0}B+\sum {\left(\kappa P_{opt}H_{\mu ,else}\right)}^{2}},$$

where *κ* denotes the efficiency of optical to electric conversion at the receivers; The noise power spectral density is *N*_0_ [A^2^/Hz], $H_{\mu ,\alpha }$ is the channel gain between user μ and LiFi AP, and $H_{\mu ,else}$ is the channel gain between user μ and the interfering LiFi AP. After modulation, at minimum half of the sub-carriers should be employed to recognize the Hermitian conjugate of the complex-valued sign. As a result, only half of the available bandwidth can be used for signal delivery in state *n*. The Shannon capacity is applied to calculate the attainable data rate between both the user *μ* and the LiFi AP α, which is stated as:

(5) $$R_{\mu ,\alpha }^{\left(n\right)}=\frac{B_{L}}{2}\;log_{2}(1+SINR_{\mu ,\alpha }^{\left(n\right)}),$$

where $B_{L}$ is the bandwidth for optical signal transmission. The time division multi-access (TDMA) approach is used in this work, and proportional decent scheduler is studied. Once proportional fairness is established, the users supplied by a LiFi AP possess an equivalent time resource, similar to [13]. Each WiFi AP in the RF cell has an Omni-directional broadcast station. In the RF system, orthogonal frequency division multiplexing access (OFDMA) is being used. The harmonic responsiveness of the channel is considered to be flattened due to low energy from mirrored routes so that all sub-carriers assigned to a particular user use the same CSI. The WiFi channel gain across users and WiFi APs is estimated as follows:

(6) $$h=\sqrt{10\frac{-L\left(d\right)}{10}\;}\left(\sqrt{\frac{K}{1+K}}h_{d}+\sqrt{\frac{1}{1+K}}h_{s}\right),$$

where *K* = 10 dB is the Rician component for indoor 60 GHz connections; *h_d_* = $\sqrt{1/2}\left(1+j\right)$ is the straight lane fading channel; *h_s_* ~ *C Ɲ* (0,1) is the distributed path fading channel; *L*(*d*) is the equivalent large-scale fading loss in decibels at the isolation range *d*, given as:

(7) $$L\left(d\right)=L(d_{0}+10\nu \;{log}_{10}\left(d/d_{0}\right)+X,$$

where in *L*(*d*_0_) = 68 dB is the benchmark path loss at *d*_0_ = 1 m; *v* = 1.6 is the route loss exponent; and *X* is the shadowing factor, which is considered to be a zero mean Gaussian distributed arbitrary variable with a standard deviation of 1.8 dB. The shadowing impact caused by human bodies near the mmWave radio connections is ignored. Each sub-carrier is believed to have equal power, and every user can be flexibly assigned sub-carriers for broadcast. In state *n*, the data rate obtained by the WiFi link across the user *μ* and the WiFi AP α can be expressed as:

(8) $$\Upsilon_{\mu ,\alpha }^{\left(n\right)}=B_{\mu }log_{2}\left(1+\frac{\lceil h_{\mu ,\alpha }^{\left(n\right)}\rceil {}^{2}P_{R}}{N_{0}B_{R}}\right)$$

where in *B_μ_* is the bandwidth given to the subscriber *μ* in the WiFi system; and $h_{\mu ,\alpha }^{\left(n\right)}$ is the WiFi channel gain across the user *μ* and the AP α according to (6). Λμ is described as the proportion of bandwidth obtained by the user *μ*. As a result, *B_μ_* can be written as:

(9) $$B_{\mu }=\Lambda_{\mu }B_{R.}$$

### 2.3. The Proposed Method

A HO develops in a dynamic system when two separate APs support a user in two neighboring states. There are no significant data received by the participants during the HO. This causes OH and spectral effectiveness impairments, which are taken into consideration in this analysis. Particularly, HOs can be categorized into four different kinds: from a WiFi AP to a LiFi AP; from a LiFi AP to another LiFi AP; and from an RF AP to another RF AP. The OH of various types of HOs in an indoor network is measured in milliseconds (ms). This OH is supposed to be significantly less than the temporal interval *Tp* from the two states.

Because of the latency in signaling engagement, the OH cannot be a fixed value in a practical system. It is thought that the Poisson distribution is an appropriate model to describe it. As a result, the OH of various types of HOs is modeled in this study as a random variable with an independent identical Poisson distribution. The probability mass function can be calculated as follows:

(10) $$\mathrm{Pr}\left(t_{ij}=x\right)=\frac{\zeta_{ij}^{x}e^{-\zeta_{ij}}}{x!},x=1,2,3\ldots$$

where *t*_*ij*_ is the OH of the AP switch from AP *i* to AP *j*, *ζ_ij_* = E[*t*_*ij*_] is the OH mean, and x is the time interval of OH measured in (ms). Because a HO reduces throughput between the AP and the user, the transmission effectiveness between two neighboring states can be expressed as follows

(11) $$\eta_{ij}=\{\begin{array}{cc} 1-\left[\frac{t_{ij}}{T_{p}}\right] & i\neq j,i,j\in C_{ℒ}\cup C_{R.}\\ 1, & i-j \end{array}$$

where the procedure [.]^+^ stands for maximum (. , 0). The product of efficiency in (10) and the interaction link data rate yields the impactful data rate with HO between each AP and user. Each user in this system is assigned to a LiFi or WiFi AP in each state. During the working period, users move in a random manner in the indoor scenario, and the APs assigned to them are changed based on their position. The CU computes the allocation result in each state while taking handover into account. Algorithm 1 depicts the evolving algorithm implemented by the CU in *N_s_* working states. Note that the core functions of the proposed technique are operating in Algorithm 1. On the other hand, the LB, HO management, and the APA are executed in Algorithm 2 wherein these are.

**Algorithm 1** The proposed TPHM-APT executed by the CU

1. Initialization: denote α′*_μ_* as the AP allocated to user *μ* in a specific state *n*;2. Input: *N_s_*, *N_u_*, *N_r_*, *N_v_*.3. Process: While *n* ≤ *N_s_* do4.               Obtain CSI for all connected users and all APs in both systems;5.               If all users’ CSI are obtained then6.                     Calculate $R_{\mu ,\alpha }^{\left(n\right)}$ and ${ϒ}_{\mu ,\alpha }^{\left(n\right)}$ according to (5) and (8);7.                     Calculate $\eta_{{\alpha^{\prime }}_{\mu }\alpha }^{\left(n\right)}$ according to (11) between each *μ* and α.8.                     Start Algorithm 2: Perform system LB, and HO management then Allocate users to APs by making allocation decisions using DRT, IAT, and UMT;9.               else10.                    Refresh and update the CSI at the CU;11.              End if12.              Update AP α′*_μ_* in the current state13.              *n* ← *n* + 1;14.Output: *M*_*β*__1,*μ*_, *U_R_*, $r_{\mu }^{\left(n\right)}$.15.              End while


An LB algorithm is presented, along with AP assignment and transmission resource allocation. In this section, the superscript (*n*) is omitted for clarity. The serving AP in state *n* − 1 for user *μ* is signified as *α′_μ_*. To wholly utilize LiFi’s spatial-spectral efficiency, users would be assigned to LiFi APs first in each state. Users who obtain decreased data rates than the threshold γ, are re-allocated to WiFi APs using the data rate threshold. The very first distribution phase employs a criterion of maximum effective throughput. The LiFi AP obtaining the greatest connectivity data rate with HO can be expressed as follows for users:

(12) $$\beta_{1,\mu }=\arg\;\max\;\eta_{\alpha_{\mu }^{\prime }}jR_{\mu ,j}.\;j\in C_{ℒ}$$

where $\beta_{1,\mu }$ is LiFi AP with the highest communication link data rate with HO for users, $\eta \alpha_{\mu }^{\prime }j$ is the transmission efficiency of the connected LiFi user in the next state *n*, and $R_{\mu ,j}$ is the achievable LiFi data rate for a user in the next state. Because each user shares the same time resource once assigned to a LiFi AP, the optical data rate for every user can be composed as:

(13) $$\Omega_{\mu }=\eta_{\alpha_{\mu }^{\prime }}\beta_{1,\mu }\;\frac{R_{\mu ,\beta_{1,\mu }}}{M_{\beta_{1,\mu }}},$$

where *M_β_*_1,*μ*_ represents the number of users supplied by LiFi AP *β*_1,*μ*_. Users who meet the condition $\Omega_{\mu }$ < *γ* are re-allocated to WiFi APs during the re-allocation phase. To meet the ADRR, the data rates of users in the WiFi system should therefore be improved. Briefly, an ideal WiFi AP is assigned to each user depending on the CSI in the present state. The criterion of maximal impactful throughput is used for AP allocation in the WiFi system, as it is in the LiFi system. The best WiFi AP for the user *μ* is displayed as:

(14) $$\beta_{2,\mu }=\arg\;\max\;\eta_{\alpha^{\prime }\mu j}\Upsilon_{\mu ,j}\;\;\Omega_{\mu }<\gamma .\;\;j\in \;C_{R}.$$

where $\Upsilon_{\mu ,j}$ is the achieved WiFi data rate for a user *μ* in the next state *n*, and the arg max probability distribution locates the maximum random variable among a set of random variables. Furthermore, an adaptive bandwidth assignment is used, which takes into account the average data rate achieved in previous states. To boost the rate of users who previously had a low average data rate, the bandwidth proportion *Λμ* can be configured as follows:

(15) $$\Lambda_{\mu }=\frac{1/\lambda_{\mu }}{\sum_{x\in U_{R}{}^{1/\lambda_{x}}}}$$

where λ*_μ_* is the median data rate for subscriber *μ* in the prior states; and $U_{R}$ is the collection of users supplied by WiFi APs in the present state. Some users’ data rate performance can be improved by employing this bandwidth apportionment procedure. As per (12) and (14), the AP assigned to subscriber *μ* in state *n* can be written as follows:

(16) $$\alpha_{\mu }=\{\begin{array}{c} \beta_{1,\mu ,\;\Omega \geq \gamma }\\ \beta_{2,\mu ,\;\Omega <\gamma } \end{array}$$

The apportioned AP *α_μ_* within every state can be ascertained using Algorithm 2. Thus, the user’s achievable data rate can be demonstrated as:

(17) $$r_{\mu }^{\left(n\right)}=\{\begin{array}{cc} \eta_{\alpha_{\mu }^{\prime }\alpha }{}_{\mu }^{\;}\frac{R_{\mu ,\alpha_{\mu }}}{M_{\alpha_{\mu }}}, & \alpha \in \;C_{R},\\ \eta_{\alpha_{\mu }^{\prime }\alpha }{}_{\mu }^{\;}\Lambda_{\mu }\Upsilon_{\mu },\alpha_{\mu } & \alpha \in \;C_{R} \end{array},$$

where *M*_α_*_μ_* represents the number of users supplied by the LiFi AP *α_μ_*. The outage probability of the QoS requirement in the hybrid system can be demonstrated using the ADRR Г as:

(18) $$\Delta =Pr\left(\frac{1}{N_{s}}\;\overset{N_{8}}{\underset{n=1}{\sum }}r_{\mu }^{\left(n\right)}<\Gamma \right).$$

where $N_{s}$ is the number of working states. According to the presented dynamic LB algorithm, the data rate criterion can have a significant impact on the likelihood of an outage.

For θ_½_ = 90° (perpendicular surface PS), the peak theoretical optical gain is obtained [41]. As a result, the optic must be in significant contact with the chip. The functioning of the optic module can be classified based on how close the receiver gain is. To achieve the greatest dynamic range, the gain must ideally be steady within the FOV.

**Figure 5:**
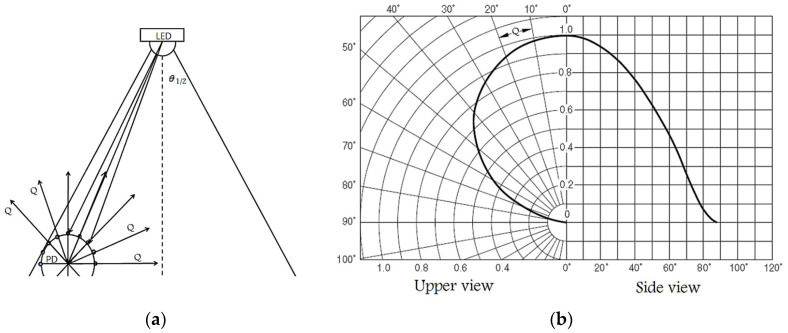
AOA based on circular PD array. (**a**) shows the radius of the PD array, and (**b**) illustration of the LED’s responsiveness.

However, the gain differs throughout the FOV, and we chose to set the FOV for the incidence threshold Ϯ to be 20° around the PD, which means that the θ will range from 70° to 110°, as shown in Figure 5a,b, wherein it will be achieved between the LiFi AP and the user determined in state *n* can be expressed as:

(19) $${Ϯ}_{\mu ,\alpha }^{\left(n\right)}=\{\begin{array}{ccc} 1, & g_{\max}\in \Theta_{F},\;P_{PD}\in \;C_{ℒ},\;0\leq \;\theta <\Theta_{F}, & \theta \leq Ϯ\\ 0, & & \theta >\Theta_{F} \end{array},$$

where the value of the Ϯ will be true (1) when the θ_½_ equals or less than 20° (ranges from 70° to 110°), otherwise it will be false (0) taking the ranges from 0° to 70°, and from 110° to 180°. The *P_PD_* is the spot of *i*-th PD defined as follows:

(20) $$P_{PD}\;(i)=RC.\;\frac{\pi }{Q-1}\times i\;(\mathrm{rad})$$

where *RC* is the radius of the spherical *PD* array (as shown in Figure 5a), Q is the path orientation from the PD, and the gain must drop rapidly outside of Ϯ as follows:

(21) $$g_{\max}=\frac{n_{1}sin\theta_{o}}{sin\theta_{a}}$$

in which *n*_1_ is the refractive indicator of the optic, $\theta_{a}$ is the predicted acceptance angle, and $\theta_{o}$ is the incident angle of the border radiation from the optic to the PD chip. Figure 5a easily identifies the Angle of Arrival AOA used to locate a light source (LED) using a circular PD array. AOA prediction is a critical technique for enabling position perception solutions, and Figure 5b depicts the LED’s responsiveness [42]. The total route paths of the PDs Qs are spread in a circular pattern with uniform angle distinctions, as shown.

In this study, user mobility is yet another essential criterion that adds value to the work. The random waypoint (RWP) mobility model [43] is a popular synthetic mobility model. The RWP requires users to continue moving in a zigzag pattern from one waypoint to the next, with the waypoints getting randomly spread. MATLAB is used to implement this random motion. *P*_WP_ and *P*_old_ are the user’s next waypoint and current location, respectively. The movement route is signified by $\vec{n}=\frac{P_{\mathrm{wp}}-P_{\mathrm{old}}}{\left|P_{\mathrm{wp}}-P_{\mathrm{old}}\right|}$. The user’s following location, which is represented by *P*_new_, is considered as follows:

(22) $$P_{\mathrm{new}}=v\tilde{t}\vec{n}+P_{\mathrm{old}}$$

where *v* signifies the user’s present velocity and $\tilde{t}$ is the time response. Whenever the user attains *P*_WP_, a fresh waypoint is chosen at random. In terms of user speed, three variants are recognized, as explained below:

1. Constant Speed CS: Each user moves at a constant speed in this mode.
2. All users’ speeds follow uniform dissemination between 0 and *v*_max_, in which *v*_max_ signifies the highest speed, which is presumed to be up to 5 m/s, and the user’s location is expected to be evaluated every 10 ms, throughout which the user could keep moving no further than 5 cm. In this work, however, the user’s location is quantified in each state *n* (as in Algorithm 2). Regarding the LiFi coverage range of 2–3 m, this configuration could even ensure a high sufficient resolution to monitor the route of motion.
3. Varying Speed VS: To make the RWP model suitable for an indoor case, the user’s speed is presumed to be constant for a quick time frame. The user’s motion during this time is referred to as an excursion. In particular, each user travels at a random speed for an arbitrary duration that is distributed evenly between 10 and 20 s. When the current excursion comes to an end, the user selects a new speed and continues moving.
4. The first two modes suppose that users are constantly on the move. Users may, however, remain static for an extended time. This is known as pausing time, and it occurs when a user completes one excursion before moving on to the next. The pausing time probability density function is presumed to be equally distributed [44]. The pausing time is arranged here to be between 0 and 10 s. There are moving users and static users in this mode at the identical time.

**Algorithm 2** Load balancing algorithm in each state for the TPHM-APT

1. Initialization: $R_{\mu ,\alpha }^{\left(n\right)}$, $\Upsilon_{\mu ,\alpha }^{\left(n\right)}$, ${Ϯ}_{\mu ,\alpha }^{\left(n\right)},$ and $\eta_{\alpha_{\mu \;}^{\prime }\alpha }$2. Process: for all users *μ* do3.                    Calculate _$\beta_{1,\mu }$ according to (12).4.                    Calculate the potential LiFi data rate Ω*μ* according to (13), user incidence angle threshold ${Ϯ}_{\mu ,\alpha }^{\left(n\right)}$ according to (19), and user movement ${ⱱ}_{\mu }^{\left(n\right)}$ according to (23).5.                    If (${Ω}_{\mu }$ ≥ γ)6.                          If (Ϯ==1)7.                                If (ⱱ ==1) then8.                                User μ is allocated to LiFi AP $\beta_{1,\mu }$.9.                                Else10.                               User μ is allocated to WiFi AP _$\beta_{2,\mu }$ according to (14).11.                   Calculate the allocated WiFi bandwidth $\Lambda_{\mu }$, according to (15)12.                               End if13.                         End if14.                   End if15.                   Calculate the average data rate per user $\lambda_{\mu }.$16.                   Calculate the achievable data rate in all the states based on (17).17.               End for


To start making the HO procedure decision quite authentic in this work scenario for portability, the user mobility threshold ⱱ for an assumed user μ in each state *n* can be expressed as:

(23) $${ⱱ}_{\mu }^{\left(n\right)}=\{\begin{array}{cc} 1, & 0\leq v<\frac{v_{\max}}{2}\\ 0, & \theta >\Theta_{F} \end{array},$$

in which the significance of the ⱱ will be true (1) once user mobility ranges between 0 and 1 m/s, indicating that the user is either not traveling (seated or waiting) or traveling slowly, otherwise the value of ⱱ will be false (0) for Algorithm 2.

The following are some highlights of the procedure preceded by both Algorithms:

- Algorithm 1 is run when the system boots up. Several fundamental tasks are carried out, such as establishing a working configuration for the system, turning on all necessary components, and locating all available access points. In this algorithm, users are counted and ranked, and then the CSI is configured based on those users’ connections to access points. After the HO and APA steps have been taken, the calculations, function calls (Algorithm 2), CSI refresh, and AP status update will take place. After the simulation time has elapsed, an output is generated as a final result. In particular, the achievable data rate between the user and the LiFi AP and the WiFi AP according to (5, 8) are factored in. Next, we compare the current state to the previous state in terms of transmission efficiency by using (11). The second algorithm, implemented as a function, will now begin, with individual users’ LBs, HOs, and APAs being handled in isolation across all states. After that, the CSI is updated, along with all of the associated AP data, such as user assignments, ADRR, and available bandwidth. The proposed scheme will work and repeat the process as long as the number of working states is smaller than the total time of simulation (n < Ns). At the end of each state, an output will occur including the number of LiFi users *M_β_*_1,*μ*_, the number of WiFi users *U_R_*, and the achieved average date rate for all users $r_{\mu }^{\left(n\right)}$ in the current state. Note that the user mobility including user speed and direction estimations are performed in Algorithm 2.
- Algorithm 2 performs the LB in each state where the three-phase HO management and APA are executed. Specifically, the achievable LiFi data rate, the achievable WiFi data rate, the IAT, and the transmission efficiency between the serving AP and the connected users are initialized. Once the process starts, the LiFi AP with the highest communication link data rate and the optical data rate is calculated according to (12) and (13). Then, the IAT value is set. The CU observes the user’s motion and provides a specification according to Equation (23). Previous research has assumed that the transmitter is completely aware of each UE’s CSI. However, accurate CSI may be relatively easier to obtain in a static condition, and from a practical perspective in the case of user mobility, obtaining the CSI is an estimation problem that cannot be error-free. Therefore, it is important to understand the effect of the channel estimation error on the system throughput in a multiuser environment. After the above calculations, the process of HO decision and APT will begin. Specifically, the optical gain data rate is being monitored (13) and compared with the pre-set DRT value, if the condition is satisfied by a user *μ*, according to (16), the user is moved to the second phase where the IAT value of user *μ* is compared and the status of the condition will be determined based on (19)–(21). The successful user *μ* from phases 1 and 2 will go through the final checking in phase 3, wherein the user mobility speed is verified and evaluated according to (22) and (23). When all three conditions are satisfied, the user will be assigned to LiFi AP where the user resides in its service area, note that using this mechanism, the targeted LiFi AP is the only option for the user to have as a potential LiFi connection and no CCI is experienced after the examination of the third phase. On the other hand, in case the user *μ* does not meet any minimum requirements for any condition throughout any of the phases, they will be assigned (or remain with) to the best available WiFi AP according to (14). Using the allocated WiFi bandwidth calculation method after assigning a user to WiFi APs according to (15), the data rate performance of some users can be enhanced. At the end of the current state, the achieved data rate of the connected user *μ*, is calculated according to (17). Finally, the average data rate of all connected users is evaluated.

**Figure 6:**
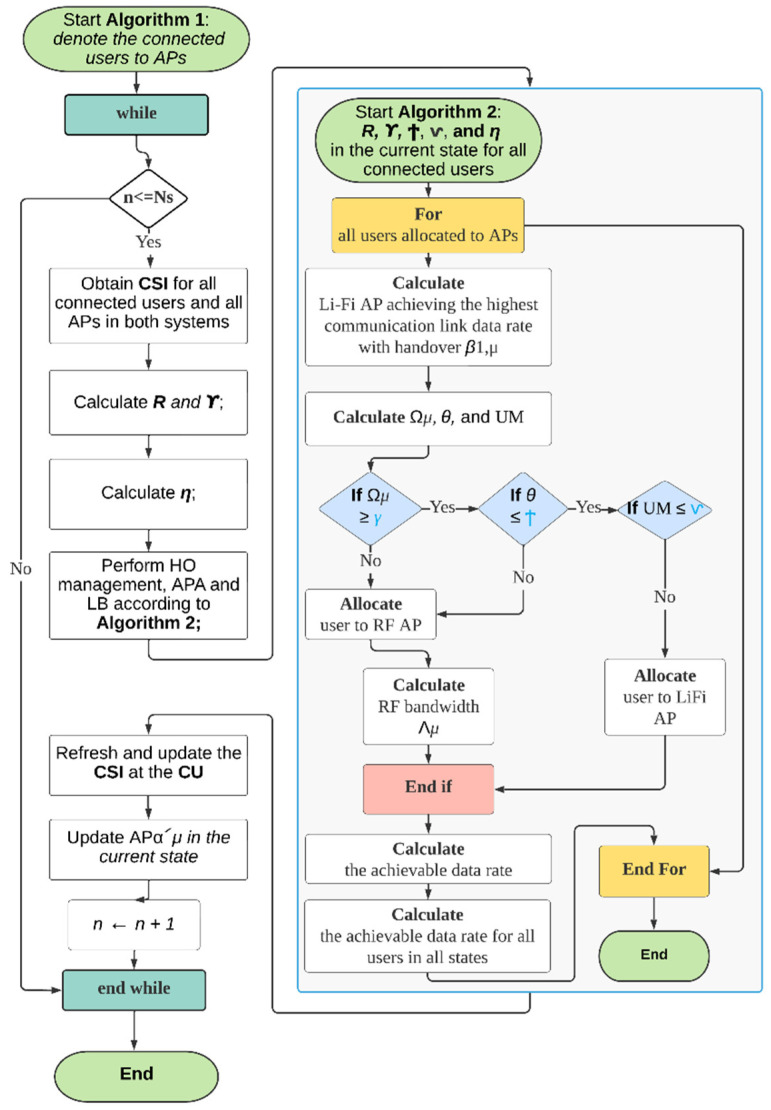
Algorithm Flowchart.

Since the system is dynamic and many changes occur in parameters and values during each state *n*, therefore, the scheme is designed to keep up with the fluctuations and changes. Note that at some point in Algorithm 1 (line 8), the LB in each state for the TPHM-APT in Algorithm 2 is called by Algorithm 1 where the main functions of HO, APA, and data rates calculations are performed. Figure 6 depicts the step-by-step procedure of Algorithms 1 and 2.

## 3. Results and Discussion

**Figure 7:**
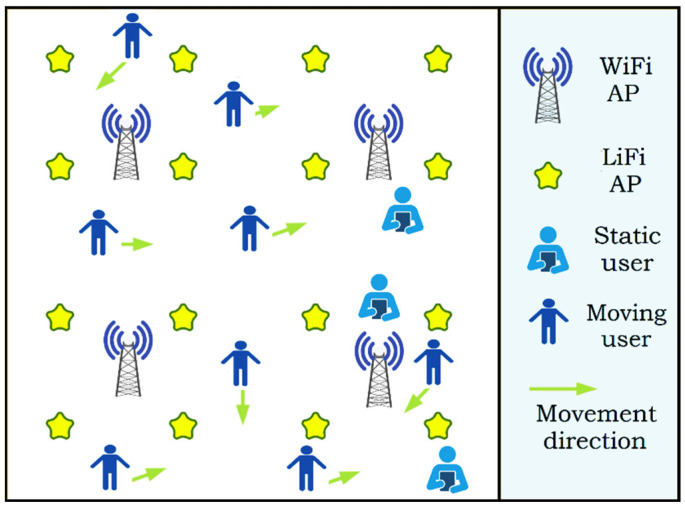
Simulation scenario.

The system configuration and modeling scenario are presented in this section. The relationship between HO locations and LiFi/WiFi throughput is also displayed, as is the variance in average throughput around stationary and traveling users. The simulated outcomes are then described, such as outage probability, HO rates, and average data rate requirement for every user. This will be accomplished by implementing Algorithms 1 and 2, which can boost productivity and reduce HO rates, as well as allocate users to the most appropriate AP in the service area. During development, we improved the system throughput that appeared as the service supplied to the user in the network. The proposed method is evaluated using Monte Carlo simulation. The simulation takes into account a 40 × 40 m open office space. This indoor scenario, displayed in Figure 7, is covered by 16 LiFi APs and 4 WiFi APs.

**Figure 8:**
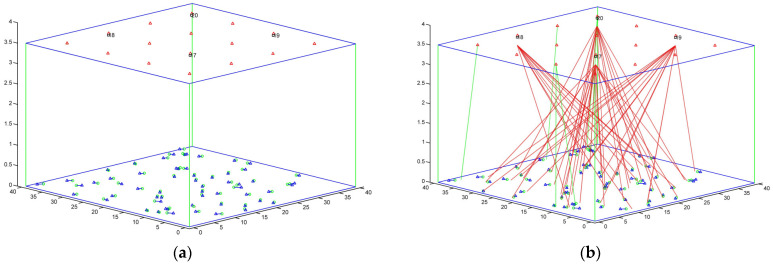

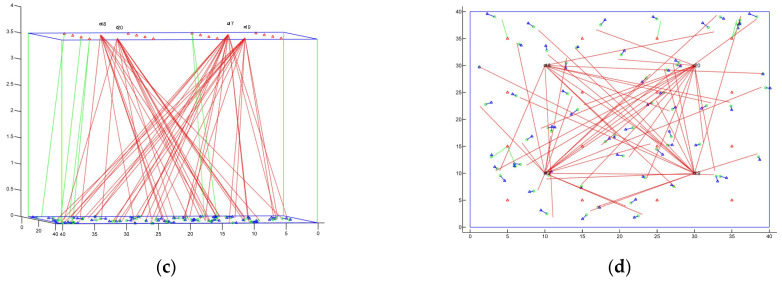
Snapshots from the simulation running (60 users). (**a**) at the start of the simulation, (**b**) during execution which shows user assignment to APs, (**c**) side view, and (**d**) upper view.

All of the LiFi APs reuse the same bandwidth, and the CCI between adjacent LiFi attocells is treated as noise. All of the users are distributed evenly and relocate at arbitrary speeds ranging from 0 to 2 m a second. Figure 8 depicts an example of simulation execution: Figure 8a depicts the beginning of the simulation, while Figure 8b depicts how users are assigned to the available APs, with the connection represented by red and green dashed lines. The red lines are WiFi signals, and the green lines are LiFi signals. Moreover, Figure 8c,d show another side view and upper view, respectively.

A HO occurs in the traditional approach when two APs are assigned to a user in adjacent states, whereas in this work, the HO is activated predicated on the three stages addressed in the methodology section, namely the receiver optical gain, receiver incidence angle, and user velocity. Following these three rules (as defined by thresholds) is anticipated to minimize HO rates, strengthen system performance, conserve network resources, and ensure higher data rates for every user. The HO overhead is Poisson dispersed in all directions. However, the HO overhead was taken into consideration in several research studies. HO overhead would inhibit the user from being relocated to another AP even if the user crossed the HO circle or met a triggering condition such as optical gain.

While considering the HO overhead for making APA decisions, several aspects, such as resource allocation and data rate prerequisites, may prompt some latency in the HO process. As a result, the proposed scheme prevents HO overhead from influencing the AP assignment process. The hybrid network requires users to have an average data rate. To study the effect of user density, a different number of users were taken into account for each result. All simulation parameters are given in Table 4.

**Figure 9:**
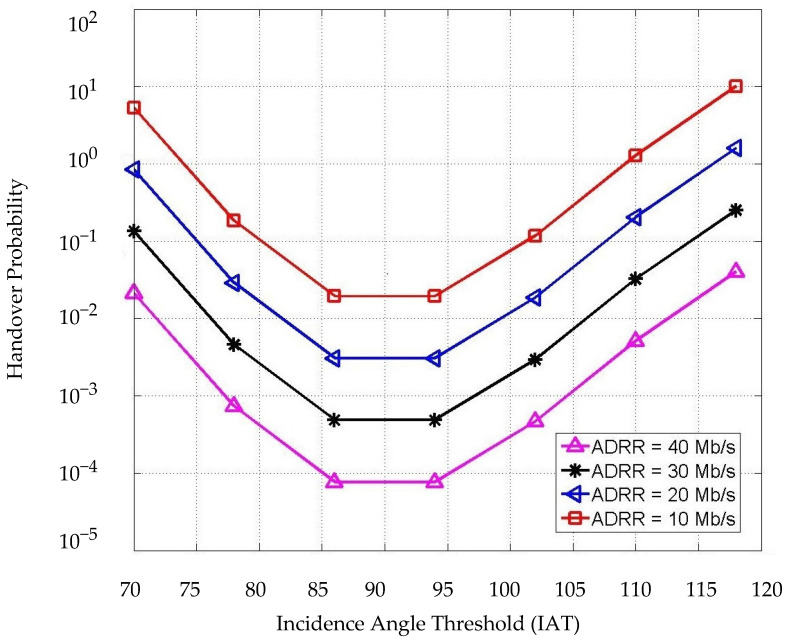
Handover Probability with the corresponding to the IAT, the expectation of the handover overhead is 25 ms.

Figure 9 shows the HO probability corresponding to the IAT with the consideration of different data rate requirement values, where the mean of the handover overhead is 25 ms. As shown, the HO probability is a convex function corresponding to the threshold Ϯ. The highest HO probability could be achieved with 10 Mb/s of data rate requirement in general, and the peak point is achieved when the IAT is set at approximately 90° for all ADRR values. In addition, it can be seen that when the value of IAT is 85°, 90°, and 95°, the value of the HO overhead tends to be more stable because the user location reached the minimum value possible under the receiver’s angle of incidence threshold Ϯ. When the threshold Ϯ < 85° and Ϯ > 95°, caused by user mobility, the HO probability starts to increase. Moreover, since the proposed scheme aims to reduce the number of users and decrease the load on LiFi APs, and re-allocate the users with lower data rate requirements to WiFi, it can be seen that different values of ADRR impact the values of HO probability besides the IAT, where lower ADRR leads to a higher probability of handover. Thus, the time resource shared by each user allocated to LiFi APs increases, so that the achievable data rate in the LiFi system becomes much higher than the ADRR for LiFi-connected users as shown in the average data rates patterns analysis later in this section.

**Figure 10:**
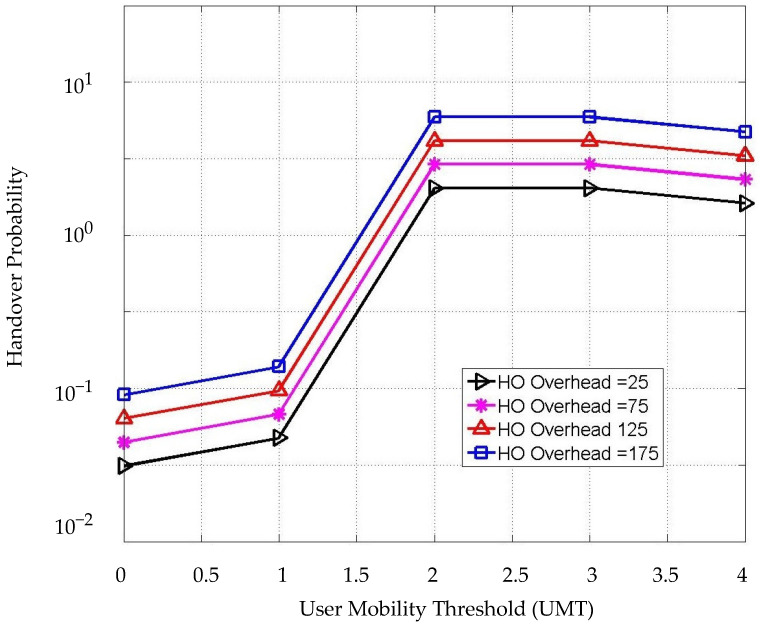
Handover probability with UMT considering different HO overhead values.

The HO probability is achieved by corresponding to the UMT with different HO overhead values as shown in Figure 10. It can be seen that the HO probability increases concerning the user mobility threshold ⱱ and the HO overhead. A 50 ms delay of HO overhead has been considered in this simulation leading to an approximately 3 dB increase of the HO probability when the UMT is 1 m/s. In addition, the HO probability increases significantly right after the speed of the user is more than 1 m/s. This process is a vital part of the decision process because it represents the third-step condition as a final check before making the HO process. The HO overhead does not have a significant impact on the HO probability in our scheme since the process focuses on the values of Ω, Ϯ, and ⱱ. When the value of ⱱ ≤ 1, the value of HO probability is still low. On the other hand, the HO probability will be high enough to trigger the HO with the increment of UMT, and it becomes stable when it reaches 2 m/s and higher, which means the user would be assigned to WiFi, and the HO is skipped. In this case, changing the value of Ω and/or Ϯ would not impact the HO decision. However, the data rates and load on each AP will dynamically be changing.

**Figure 11:**
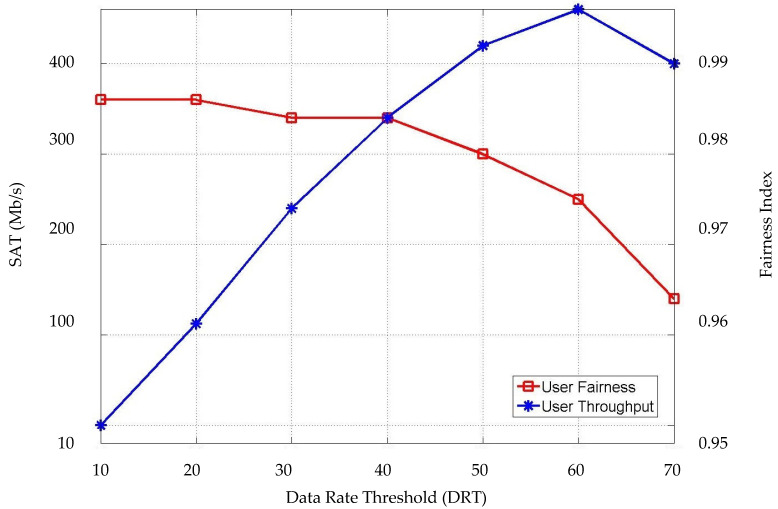
Average system throughput and fairness index with DRT (handover overhead 25 ms).

In Figure 11, the average system throughput (SAT) and user fairness are considered concerning the data rate threshold (DRT). Here, a fairness index is applied to evaluate user fairness [13], which can be expressed as:

(24) $$I=\frac{{\left(\sum_{i=1}^{i=N}x_{i}\right)}^{2}}{N\sum_{i=1}^{i=N}\;x_{i}^{2}}$$

where *I* is the fairness index, $x_{i}$ is the achievable data rate of each user, and *N* is the number of users. The fairness index is a fractional value between 0 and 1, where 1 represents perfect user fairness. As shown in Figure 11, the DRT has an effect on the SAT and the fairness index (FI). When the value of *γ* increases, the overall system throughput decreases, this is because it is related to the user data rate requirement, which means fewer users are allowed to be served by LiFi APs. An optimal FI is achieved when the DRT is set to 60 M/s. The value of the FI decreases monotonically when *γ* > 40 M/s. On the other hand, the value of the SAT reaches its best with a *γ* of 10 and 20 M/s. When the value of *γ* > 60 M/s, the throughput starts going down because the higher data rate requirement makes it difficult to serve more users with high data rates, which leads to serving fewer users per LiFi AP where the load on the LiFi resources would be decreased as planned. Specifying the value of DRT in the system is important for controlling the load on the system besides considering the number of users.

The above results consist of showing the relationship between the three thresholds used in the proposed scheme with the HO probability and SAT. Specifically, the IAT and UMT were linked to the HO probability, which is the main aim of this study, because those two thresholds represent the second and the third phases of our three-stage process. On the other hand, the first threshold DRT was linked to the system throughput and FI to show the system performance.

Additional data were gathered during the simulation process for investigating the user density and HO rates. The data were analyzed using excel sheets. To evaluate and study the effect of user density on the system performance, four cases were taken into account as follows:

1. Case 1 consists of 1 user;
2. Case 2 consists of 15 users;
3. Case 3 consists of 30 users; and
4. Case 4 consists of 60 users.

**Figure 12:**
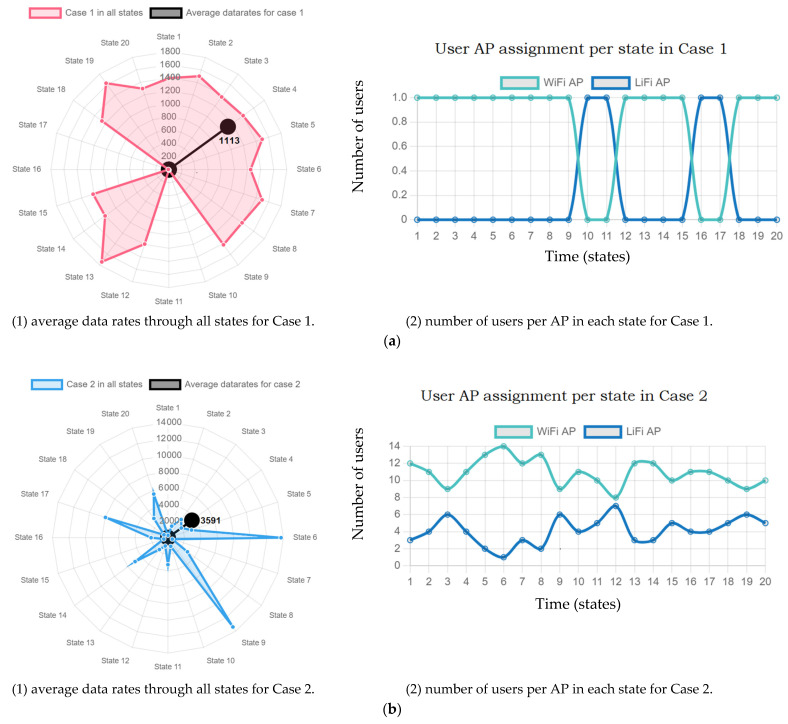

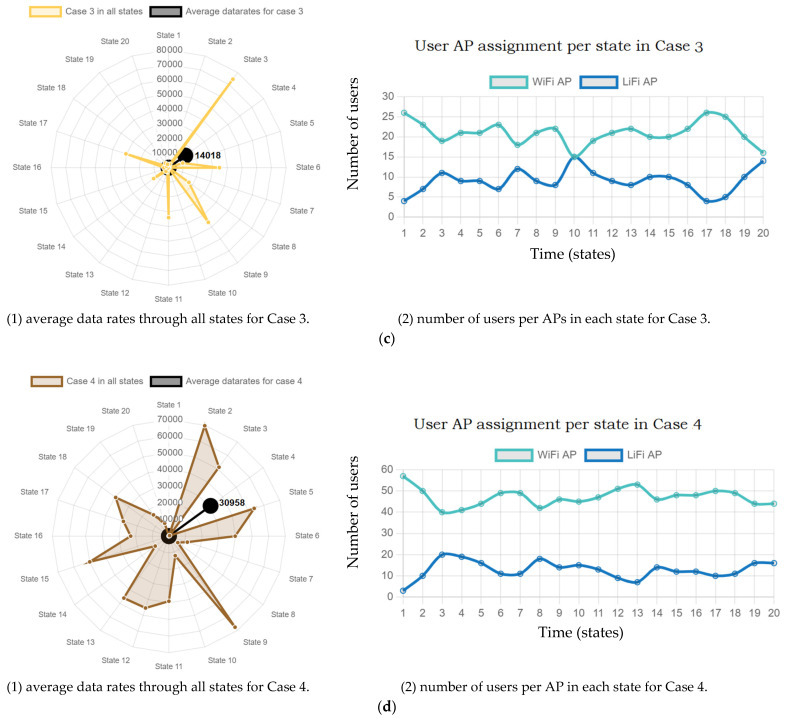
Theoretical results of average data rates patterns, user density, and user assignment per AP. (**a**) Case 1, (**b**) Case 2, (**c**) Case 3, and (**d**) Case 4.

The following Figure 12 shows the overall data rates in each state for all connected users while taking into account 16 LiFi APs and 4 RF AP. As mentioned earlier, the total simulation time is 60 s, which equals 120 working states *n*. For the user density analysis, only 20 states *n* (10 s) were taken for user density impact investigation for simplicity. As shown in Figure 12a(1), the highest data rate achieved is 1741 M/s in state number 19. On the other hand, the lowest rates were 5.7 Mb/s, 7.3 Mb/s, 9 Mb/s, and 9.6 Mb/s in states 10, 11, 16, and 17, respectively. This indicates four handovers in four states out of 20 states as shown in Figure 12a(2), where LiFi AP was assigned only four times for one connected user. In addition, the data rates in those four states refer to the lowest value of DRT, and according to Figure 9, the highest HO probability that could be achieved was at 10 Mb/s or less of data rate requirement. Moreover, as Figure 11 shows, the lowest throughput was with DRT of 10 compared with 60 of DRT. In addition, the highest user fairness was achieved with a DRT of 10 because when the requirement of data is low, more users can be satisfied.

Figure 12b presents Case 2 data. It shows the average data rate of 15 users where the highest value achieved was 13,710 Mb/s (state 6) and 13,391 Mb/s (state 9) for one and six LiFi users, respectively. On the other hand, the lowest data rate achieved was 288 Mb/s (state 1) and 551 Mb/s (state 7) for three LiFi users each. Various factors are involved in this dynamic environment such as: (i) user location, (ii) user distance to APs, (iii) user data rate requirement, (iv) user velocity, (v) UE, (vi) optical gain, (vii) UE receiver FOV, (viii) the angle of incidence, (ix) network resources, (x) HO overhead, and others. This leads to variations of average data rates with a different number of LiFi users, for example, the number of LiFi users in state number 12 are 7 achieved 1060 Mb/s of average data rate. Furthermore, there were four users connected to LiFi in two consecutive states including state numbers 16 and 17 but achieved different data rates including 2044 Mb/s and 7968 Mb/s, respectively.

Figure 12c presents Case 3, the highest data rate achieved was 74,457 Mb/s during the third state, while the lowest data rate was 114 Mb/s during the seventh state. Finally, in Figure 12d, the highest data rate was 69,716 Mb/s during the second state, whereas the lowest was 86 Mb/s in the first state. In addition to all the aforementioned results, the average data rate for the total time (20 states) was 1113 Mb/s in Case 1, 3591 Mb/s in Case 2, 14,018 Mb/s in Case 3, and 30,958 Mb/s in Case 4.

After taking careful observations from the results above, it can be seen that the lowest number of the assigned LiFi users was as follows: (i) Case 2: one user in the sixth state for one time only, (ii) Case 3: four users twice in the 1st and 17th states each, (iii) Case 4: three users in the first state for one time only. Furthermore, the highest number of the assigned LiFi users is as follows: (i) Case 2: seven users in the 12th state for one time only, (ii) Case 3: 15 users in the 10th state for one time only, and (iii) Case 4: 20 users in the third state for one time only, and that is almost 50% of total users for Cases 2 and 3, while it forms 33% for Case 4.

The shapes of Figure 12a(2),b(2),c(2),d(2) are all in symmetric shape since the nature of the working system because it presents the assigned users to LiFi and WiFi APs in a trade-off style that expresses the HO rates, where every time a LiFi user is experiencing a HO it will be transferred to the WiFi AP, which shows an incrementing number of users in opposite to a decrement of the other network.

Handover rates were calculated and analyzed according to simulation results, the HO rate results include the number of HO in each state in every case, and the total and average number of HO as well. Furthermore, the percentage of HO is shown to accurately evaluate our scheme using the following expression:

(25) $$\mathrm{HO}\;\mathrm{rate}=\frac{\mathrm{Achieved}\;\mathrm{HO}\;\mathrm{per}\;\mathrm{state}}{\mathrm{Total}\;\mathrm{number}\;\mathrm{of}\;\mathrm{HO}}\times 100\%,$$

**Figure 13:**
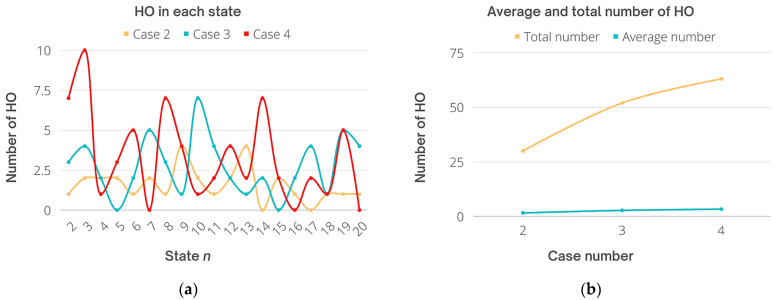
Statistics of HO rates. (**a**) Number of HO in each state, and (**b**) Total and the average number of each case.

**Figure 14:**
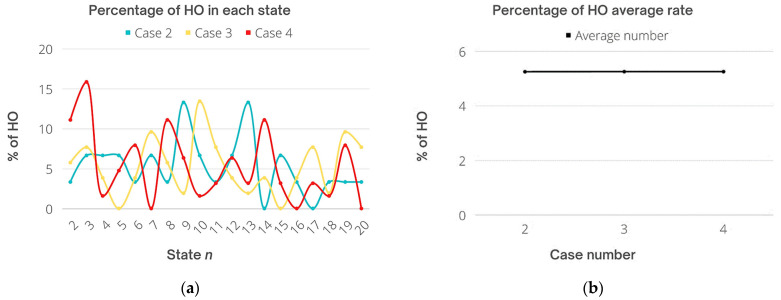
Percentage of HO rates. (**a**) Throughout each state, and (**b**) Total and the average number of each case.

Based on the obtained output including the number of users in each state throughout the total time of simulation, the average and total HO rates were calculated as shown in Figure 13. Specifically, Cases 2, 3, and 4 are taken into account for HO rates. The HO number during the second case for 15 users was very low. Specifically, most handovers were for one or two users in each state, which kept the rate low as possible for this dynamic system. The highest number of users who experienced a handover was four at most in two states only, while in other states, the handover rate was 0 for two times only. For Case 3, the frequent number of transferred users was around two, three, or four users mostly, and the highest number of users who faced HO was seven users in one state only, and zero handovers took place two times as well. Finally, in Case 4, the highest number of HO was for 10 users at one time, and the frequent number of users transferred was one, three, four, and five mostly, while zero HOs took place three times. Moreover, throughout 20 states *n*, the average number of transferred users was 1.57, 2.73, and 3.31 for Cases 2, 3, and 4, respectively. On the other hand, the total HO number was 30, 52, and 63 times. It can be seen that the total HO of Case 2 has been reduced compared with Case 4 considering the user density, since the number of users increases the scheme aims to reduce the HO rates to preserve resources and keep the system stable. This is because the chances of users to fulfill the conditions of three stages to be assigned to LiFi are getting more difficult including optical gain threshold, narrow receiver FOV in a tight area, and user speed. Derived from Figure 13 results, Figure 14 shows the percentage of HO rates for users, where the highest percentage was 13.3%, 13.46%, and 15.87% for Cases 2, 3, and 4, respectively. Note that for Cases 2 and 3, the rate is almost the same, whereas for Case 4 it is slightly increased.

Finally, the percentage of the average of all cases was 5.25% for Case 2, 5.25% for Case 3, and 5.25% for Case 4. Eventually, the average percentage for all cases is the same obtained by the proposed scheme which aims to not only prevent unnecessary handovers that could degrade the performance of the network but to reduce the overall HO rates based on the user data rate requirements and HO overhead.

## 4. Conclusions

A novel three-phase handover management and access point transition TPHM-APT was designed and developed in this study that aims to control and reduce HO rates for connected users in addition to access point assignment and load balancing in the hybrid LiFi/WiFi network. This research focuses on APA and HO problems with consideration of user mobility and user density. Former research used SINR, average data rate, RSS, user velocity, and/or optical gain, whereas the scheme in this research aims to use a combination of factors in addition to receiver FOV and angle of incidence to make decisions for HO process and APA that is used to allocate a user to either a LiFi or WiFi APs. The goal of this process would decrease the load on the LiFi resources and increase system stability. The performance analysis of the proposed scheme was carried out using Monte Carlo simulations and mathematical analysis. Four cases were taken into consideration, each case consisted of a different number of users for a more accurate performance evaluation. The results show reduced HO rates during all cases, high throughput, and stable connection for all users. It was also shown that the percentage of average HO in all cases is the same, and that means the HO rates would be similar when using the proposed scheme even when the number of users increases.

## Figures and Tables

**Table 1:** Summary of related studies and our study.

Ref.	Year of Pub.	Description	Presented Results and Aims (Plots and Graphs)	Factors Considered									
				Problems					Others				
				Mobility	Interference	User Density	Load Balancing	HO Overhead	APA/APS	Implementation	Handover Type	Topology	Transmission Dir.
[32]	2006	To provide users with enhanced quality of service (QoS). A brand-new fuzzy-logic (FL)-based decision-making algorithm for VHO was proposed as a solution to the LOS blockage problem. This algorithm is capable of integrating the advantages of both approaches to deliver good HO in terms of packet transfer time.	Average transfer delay, failure probability of HO to radio link, and VHO execution delay.	✓	✕	✕	✕	✕	✕	S	VHO	Hybrid Radio-Optical	NS
[16]	2015	User roaming and HO signaling OHs were taken into consideration while suggesting a flexible LB system.	CDF of the user data ratio, CDF of the distance between the LiFi APs and the handover location, and spatial throughput.	✓	✓	✕	✓	✓	✓	S	VHO and HHO	Hybrid LiFi-WiFi	DL
[19]	2015	To address the primary LB issue. Algorithms for both centralized and decentralized resource allocation were used to construct cooperative LB that achieves proportional fairness (PF).	Average user throughput with LOS blocking probabilities.	✕	✓	✓	✓	✕	✓	S	HHO	Hybrid VLC-WiFi	DL
[26]	2015	This study suggested two different VLC HO mechanisms since a suitable HO mechanism needs to be created for VLC to be an entire inside solution.	Provide a higher data rate for both the overall system and individual users in the HO region.	✓	✓	✕	✕	✕	✕	S	HHO	VLC	NS
[29]	2015	Developed a Markov decision process model of the VHO decision-making procedure and used a dynamic technique to achieve a trade-off between shifting costs and latency requirements.	Packet loss rate, average delay, average queue length, and number of VHO.	✕	✕	✕	✕	✕	✕	S	VHO	Hybrid VLC-RF	BiL
[30]	2015	To minimize the VHO process’s signaling costs. Due to fluctuating traffic and network situations, mobility control was taken into consideration. Based on two VHO techniques, the authors proposed a VHO algorithm through prediction (PVHO) (IVHO and DVHO). Each time a disruption occurs, PVHO determines the ideal dwell time, estimates the efficacy of both schemes, and selects the more effective one.	Average transfer delay, the delay performance of the algorithms with average message sizes, and the delay performance with average interruption duration.	✓	✕	✕	✕	✕	✕	S	VHO	Hybrid VLC-LTE	BiL
[17]	2017	A proposed two-stage APS approach. The users who should be connected to WiFi are first identified using a fuzzy logic approach. Second, the remaining users are allocated in a homogenous LiFi network setting. Iterations are not necessary for the suggested solution, which uses a centralized algorithm, to obtain a stable state with low-power computing.	User throughput vs. computational complexity and the number of users, system performance vs. the number of users, user’s throughput vs. average required data rate, and user’s throughput vs. the number of WiFi channels.	✕	✓	✓	✕	✕	✓	MCS	✕	Hybrid LiFi-WiFi	DL
[22]	2017	Users who are encountering significant blockages might switch to the RF system to increase their data rate. To simulate a real-world communication setting, this study defined blockages, the user data rate demand, and the random alignment of LiFi receivers. For hybrid LiFi/RF networks, a novel LB strategy based on EGT has been developed to enhance user QoS.	Ratios of EGT payoffs to the global optima, the average user QoS corresponding to the iteration number, evaluation of user QoS with different RF setups including data rate requirement. the user QoS with maximal vertical ROA, the CDF of user QoS, and the average data rate, average user QoS, and CDF of user data rate with different blockage densities.	✕	✓	✓	✓	✕	✓	S	✕	Hybrid LiFi-RF	NS
[23]	2017	They concentrated on complex multi-LED APS strategies and developed a multi-armed bandit approach to assist decisions on wisely choosing APs by utilizing the strength of online learning algorithms.	The normalized throughput of the selected VLC link, the system’s total accumulated normalized throughput, and the decision probability distribution.	✕	✓	✕	✕	✕	✓	MS	✕	Hybrid LiFi-WiFi	BiL
[20]	2018	The power of each AP is divided among the connected users according to an optimization problem designed to maximize the maximum total possible data rate. Proposed a novel, effective method that, after constructing optimal dual variables in the context of one another, determines one or the other.	System capacity, system fairness, and convergence.	✕	✓	✓	✓	✕	✕	MCS	✕	Hybrid VLC-RF	NS
[28]	2018	For LiFi networks that included two LiFi APs and an RF AP, a received signal strength indicator (RSSI)-based HO mechanism was taken into consideration to evaluate the HO probability of the UE with arbitrary orientations.	The HO and association probabilities, and The normalized average number of HO.	✕	✕	✕	✕	✕	✕	MCS	VHO	Hybrid LiFi-RF	DL
[21]	2019	Since the coverage areas of the various networks overlap, APS becomes difficult to implement. It was suggested to use single transmission (ST) and multiple transmission (MT) modes for mobility-aware load balancing (MALB).	Average cell dwell time of mobile users for different network scales, rates of HHO and VHO, system fairness vs. the number of users, system throughput vs. the number of users, user speed, computational complexity, and occurrence rate of blockages, and HO rate for different random waypoint RWP modes.	✓	✓	✓	✓	HHO overhead	✓	MCS	VHO and HHO	Hybrid LiFi-WiFi	DL
[36]	2019	Based on reference signal received power (RSRP), a novel HO skipping strategy was presented. To obtain the HO target, the new method combines the value of RSRP and its rate of change. There is no need for more feedback on the suggested approach.	HO rate, coverage probability, and throughput vs. user speed, coverage probability vs. threshold SNR, HO rate vs. the occurrence rate, and throughput vs. the weight coefficient.	✓	✕	✕	✕	HHO overhead	✕	MCS	HHO	LiFi	NS
[24]	2020	An AP assignment technique that maximizes long-term system throughput while providing the necessary user fairness and satisfaction was developed using a reinforcement learning (RL) algorithm. With regular and non-uniform distributions of users, two distinct scenarios relying on the random waypoint model have been investigated.	Computational complexity, user satisfaction, user Satisfaction with RWP, capacity outage probability, and capacity outage probability with RWP.	✓	✓	✓	✓	✕	✓	S (python)	✕	Hybrid LiFi-WiFi	DL
[25]	2020	An innovative HO mechanism that uses machine learning to implement a dynamic coefficient to change the choice of LiFi or WiFi has been proposed. To create HO decisions, the proposed approach weighs channel reliability, resource accessibility, and user mobility. Through ANN, this coefficient is taught for various scenarios.	Average achievable throughput vs. the user’s speed, and handover rates of HHO and VHO.	✓	✓	✕	✕	✕	✓	S	VHO and HHO	Hybrid LiFi-WiFi	NS
[38]	2021	Analyzes the cross-tier HO rate between the primary and secondary cells while presenting a two-tier LiFi network. Closed-form formulas for the cross-tier HO rate, ping-pong frequency, and sojourn duration in respect of the acquired optical signal strength, time-to-trigger, and user mobility were developed using stochastic geometry.	P2S handover rate, P2S ping-pong rate, and average sojourn time inside a secondary cell by analyses and simulation.	✓	✕	✕	✕	✕	✕	S	HHO	LiFi	NS
This work	Proposes TPHM-APT scheme for dynamic LB. Aims to control and reduce HO rates, and ensure system high throughput and system stability by taking user mobility, user density, optical gain, receiver FOV, and user speed into consideration.		Handover probability vs. IAT, Handover probability vs. UMT, Handover probability vs. DRT, System average throughput, User density, User fairness, Average and total HO rates.	✓	✓	✓	✓	✓	✓	MCS	VHO and HHO	Hybrid LiFi-WiFi	DL
Terms	S: simulation; MCS: Monte Carlo simulation; MS: MATLAB simulation; H: hybrid; NA: not specified; DL: downlink; UL: uplink; BiL: Bidirectional link;												

**Table 2:** Hardware specifications of the device used for the simulation.

Hardware	Specification
CPU	Intel^®^ Core™, Lenovo, China, i5-7200 U CPU @ 2.50 GHz 2.70 GHz
RAM	8.00 GB DDR3
Storage	HDD 1 TB SSD Lenovo
Operating System	Microsoft Windows 10., 64-bit, x64-based processor.

**Table 3:** Notations and symbols in this study.

Term/Notation	Meaning	Appears in Eq.	Term/Notation	Meaning	Appears in Eq.
*N_v_*	The number of LiFi APs	-	*v*	The path loss exponent	(7)
*N_r_*	The number of WiFi APs	-	$\Upsilon_{\mu ,\alpha }^{\left(n\right)}$	The data rate achieved by the WiFi link between user *μ* and WiFi AP α	(8), (14) and (17)
*N_u_*	This is denoted as the number of the users	-	*B_μ_*	The bandwidth allocated to user *μ* in WiFi system	(8) and (9)
*N_s_*	The number of the working states	-	$h_{\mu ,\;\alpha \;}^{\left(n\right)}$	The WiFi channel gain between user *μ* and AP α in state *n*	(8)
*C* ℒ	The set of optical attocells	-	$P_{R}$	The power consumption constraints for WiFi APs	(8)
*C Ʀ*	The set of WiFi cells	-	$B_{R}$	WiFi bandwidth	(8) and (9)
ϰ_DC_	A DC bias voltage source	-	Λ *_μ_*	The proportion of the bandwidth that user *μ* obtains from WiFi	(9), (15) and (17)
α	Connected LiFi AP	-	Pr	The probability mass function	(10) and (18)
*μ*	A given user	-	*t*_*ij*_	The OH of the AP switch from AP *i* to AP *ij*	(10) and (11)
θ _1/2_	The half-intensity radiation angle	(1)	ζ_*ij*_	The mean of the OH.	(10)
ϕ	The angle of irradiation	(1)	$\eta_{ij}$	The transmission efficiency between two neighboring states	(11)–(14) and (17)
θ	The angle of incidence	(1) and (2)	T *_p_*	Interval time	(11)
Θ*_F_*	The half angle of the receiver’s FOV	(1) and (2)	*β*_1,*μ*_	LiFi AP with highest communication link data rate with HO for users	(12), (13) and (16)
*H*	The optical channel gain of a line of sight (LoS) channel	(1) and (4)	Ω*_μ_*	The optical data rate for LiFi users	(13), (14) and (16)
*m*	The Lambertian index	(1)	*M* *β*_1,*μ*_	The number of users served by LiFi AP *β*_1,*μ*_	(13) and (17)
A *_p_*	The physical area of the receiver photo-diode	(1)	*β*_2__,*μ*_	The optimal WiFi AP for user	(14) and (16)
z	The horizontal distance from a LiFi AP to the optical receiver	(1)	γ	Threshold of data rate	(14) and (16)
h	The height of the room	(1)	λ *_μ_*	The average data rate in the previous states for user *μ*	(15)
g *(* θ *)*	The concentrator gain	(1) and (2)	*U* *_R_*	The set of the users served by WiFi APs in the current state.	(15)
*T* *_p_*	A state where all of the users receive the allocation results from the CU and receive signals from APs with constant data rates	(1)	α *_μ_*	The AP allocated to user *μ* instate *n*	(16)
*T_s_(* θ *)*	The gain of the optical filter	(1)	$r_{\mu }^{\left(n\right)}$	The achieved data rate of all users	(17) and (18)
x	The refractive index	(2)	∆	The outage probability of the QoS requirement	(18)
ı	Average optical power to optical power conversion	(3) and (4)	Г	The average data rate requirement	(18)
*P*_opt_	The average transmitted optical power of LiFi AP	(3) and (4)	Ϯ	Threshold of incidence angle	(19)
*P_t_*	The electric power of the signals	(3)	*P_PD_*	The position of *i*-th PD	(19) and (20)
SINR	The signal-to-interference-plus-noise ratio	(4) and (5)	$g_{\max}$	Gain drops outside the Ϯ	(19) and (21)
*к*	The optical to electric conversion efficiency at the receivers	(4)	*RC*	Denotes the radius of circular PD array	(20)
*N*_0_	The noise power spectral density	(4)	*n*_1_	Denotes the optic’s refractive index	(21)
*B*	Bandwidth	(4)	$\theta_{a}$	Is the anticipated acceptance angle	(21)
$R_{\mu ,\;\alpha \;}^{\left(n\right)}$	The achievable data rate between user *μ* and LiFi AP *α*	(5), (12), (13) and (17)	$\theta_{o}$	Describes the incident angle of the boundary rays from the optic to the PD chip	(21)
*B* *_L_*	The bandwidth for optical signal transmission	(5)	*v*	The user’s current speed	(22) and (23)
*h*	The WiFi channel gain between users and RF APs	(6)	$\tilde{t}$	The time resolution.	(22)
*K*	The Rician factor for indoor WiFi channel	(6)	$\vec{n}$	The movement direction	(22)
*h_d_*	The fading channel of the direct path	(6)	*P*_WP_	The user’s next waypoint	(22)
*L*(*d*)	The corresponding large-scale fading loss at the separation distance *d*	(6) and (7)	*P*_old_	The user’s current position	(22)
*h_s_*	The fading channel of the scattered path	(6)	P_new_	The user’s next position	(22)
*X*	The shadowing component	(7)	ⱱ	Threshold of user mobility	(23)

**Table 4:** Simulation Parameters.

Parameters	Value
Number of users	1, 15, 30, and 60
Simulation area	40 m × 40 m
Number of WiFi AP	4
Number of LiFi AP	16
Mobility model	RWP
Room height	3.5 m
Optical energy per LiFi AP	9 W
Modulation bandwidth of LiFi AP lamp	40 MHz
PD size	1 cm^2^
Radiation angle half-intensity	60
Optical filter gain	1.0
FOV semi-angle of the receiver	90
Refractive index	1.5
RF transmitter energy per AP	1 W
RF transmitted bandwidth per AP	20 MHz
Interval of each state	0.5 s
Simulation time	1 min

## Data Availability

Not applicable.
